# How unmeasured muscle mass affects estimated GFR and diagnostic inaccuracy

**DOI:** 10.1016/j.eclinm.2020.100662

**Published:** 2020-12-01

**Authors:** Brian J. Nankivell, Lachlan F.J. Nankivell, Grahame J. Elder, Simon M. Gruenewald

**Affiliations:** a Departments of Renal Medicine, Westmead Hospital, Westmead, Australia; b University of Sydney, Australia; c Nuclear Medicine, Westmead Hospital, Westmead, Australia

**Keywords:** DEXA, eGFR, Diagnostic accuracy, Kidney transplantation, ASMI, appendicular skeletal muscle index, AUC, area under the curve, BMI, body mass index, BSA, body surface area, CG, Cockcroft-Gault (eGFR estimated from creatinine clearance), CKD-EPI, Chronic Kidney Disease EPIdemiology (eGFR formula), CV, coefficient of variation, DEXA, dual-energy x-ray absorptiometry, eGFR, estimated GFR (implying a creatinine-based formula), GFR, glomerular filtration rate, MDRD, Modification of Diet in Renal Disease (eGFR formula), mGFR, measured GFR (using a reference method), NPV, negative predictive value, PPV, positive predictive value, ROC, receiver operating characteristic, Tc^99m^ DTPA, Technetium-99 m diethylene-triamine-pentaacetic acid

## Abstract

**Background:**

Estimated glomerular filtration (eGFR) results based on serum creatinine are frequently inaccurate with differences against measured GFR (mGFR) often attributed to unmeasured non-functional factors, such as muscle mass.

**Methods:**

The influence of muscle mass (measured by dual-energy x-ray absorptiometry, DEXA) on eGFR error (eGFR-mGFR) was evaluated using isotopic mGFR (Tc^99m^ DTPA plasma clearance) in 137 kidney transplant recipients. Serum creatinine was measured by isotopic-calibrated enzymatic analysis, converted to eGFR using Chronic Kidney Disease EPIdemiology (CKD-EPI) formula, then unindexed from body surface area.

**Findings:**

Unindexed CKD-EPI eGFR error displayed absent fixed bias but modest proportional bias against reference mGFR. eGFR error correlated with total lean mass by DEXA (*r*=-0·350, *P*<0·001) and appendicular skeletal muscle index (ASMI), a proxy for muscularity (*r*=-0·420, *P*<0·001). eGFR was falsely reduced by -5·9 ± 1·4 mls/min per 10 kg lean mass. Adipose mass and percentage fat had no effect on error. Muscle-associated error varied with each eGFR formula and influenced all CKD stages. Systemic eGFR error was predicted by ASMI, mGFR, recipient age, and trimethoprim use using multivariable regression. Residual plots demonstrated heteroscedasticity and greater imprecision at higher mGFR levels (*P*<0·001), from increased variance corresponding to higher absolute values and unreliable prediction by serum creatinine of high mGFR. Serum creatinine correlated with ASMI independent of mGFR level (*r* = 0·416, *P*<0·001). The diagnostic test performance of CKD-EPI eGFR to predict CKD stage 3 (by mGFR) was weakest in cachexia (sensitivity 68·4%) and muscularity (specificity 47·4%, positive predictive value 54·5% for the highest ASMI quartile).

**Interpretation:**

Serum creatinine and eGFR are imperfect estimates of true renal function, with systemic errors from muscle mass, tubular secretion, and intrinsic proportional bias; and additional inaccuracy at the extremes of renal function and patient muscularity. Cautious interpretation of eGFR results in the context of body habitus and clinical condition is recommended.

**Research in context**

**Evidence before this study**

eGFR values derived from serum creatinine are frequently inaccurate when compared against GFR reference methods. The cause of this inaccuracy is likely related to non-functional factors, including unmeasured muscle mass and tubular secretion of creatinine. Accurate data are sparse.

**Added value of this study**

Pervasive systemic error in eGFR results due to muscle mass was present across all mGFR levels. Error against isotopic reference GFR was substantial at −5·9 ± 1·4 mls/min for every 10 kg of lean muscle mass.

The muscle-associated eGFR error was dependent on the formula used for calculation: CKD-EPI was an improvement over the older MDRD formula. eGFR error was greatest in cachexia (sensitivity 68.4% to predict CKD 3) and muscular patients (specificity 47.4%, PPV 54.5% for the highest ASMI muscularity quartile). Test performance was poor at the extremes of body habitus.

Inaccuracy and imprecision of eGFR at high function (known from studies, cause unknown) was due to a mathematical effect on variance, muscle mass error, and a predictive failure of creatinine as a biological marker.

Serum creatinine was a suboptimal marker of GFR at normal functional levels (observed in population studies, but without clear cause). Our data demonstrated that creatinine input from muscularity was the main contributor of serum creatinine concentration at normal GFR levels (rather than functional renal clearance).

**Implications of all the available evidence**

Millions of automated eGFR reports are delivered daily to clinicians at point-of-care along as a marker of renal function. Many are inaccurate. eGFR based on serum creatinine is an imperfect estimate of true renal function because of intrinsic biology and unrecognized contribution of muscular input. Cautious interpretation of eGFR results is recommended.

Alt-text: Unlabelled box

## 1 Introduction

Nephrologists spend a considerable proportion of their professional careers pondering the meaning of serum creatinine results. Creatinine is the organic nitrogenous by-product from non-enzymatic conversion of phosphocreatine; the primary dispatchable energy source for contracting skeletal and myocardial muscle cells. Serum creatinine concentration reflects the balance of total input from muscle mass and diet against renal excretion (including tubular secretion of 10–15%). Very little creatine and creatinine are metabolized in kidneys, muscle, liver, and pancreas [1]. Creatinine is convenient, inexpensive to measure, and widely available. Technical improvements including mitigation of non-creatinine chromogens from the Jaffe reaction, introduction of enzymatic methods and isotopic standardization of creatinine have increased accuracy and measurement precision. Because creatinine is small (113 Da), water-soluble, non-protein bound, and freely filtered across the glomerulus without significant tubular reabsorption, it is the principal endogenous indicator of glomerular filtration rate (GFR) used in clinical practice.

Because direct measurement of GFR is laborious and expensive to undertake, estimated GFR (eGFR) equations were developed from easily measurable markers including serum creatinine, to provide rapid, repeatable and inexpensive estimations of kidney function. Serum creatinine is converted into a clinically meaningful value by mathematical transformation [2], and scores of eGFR formulae have now been published [3], [4], [5], [6]. The Chronic Kidney Disease EPIdemiology collaboration (CKD-EPI) formula [5] has supplanted the older Modification of Diet in Renal Disease (MDRD) formula [4]. Automated eGFR reports now accompany serum creatinine results at point-of-care and are widely used for detection of chronic kidney disease, patient management, and research. However, eGFR values derived from serum creatinine are frequently inaccurate when compared against measured GFR (mGFR) reference methods. Only 24–38% of eGFR results typically fall within the clinically relevant P_10_ accuracy standard (proportion absolute percentage error <10%) [6]. Disease misclassification by eGFR are well-known pitfalls to experienced nephrologists. Examples include false underestimation of GFR in young muscular men (incorrectly labelled as renal failure), or overestimation in frail, sarcopenic women (underestimating CKD with near normal creatinine).

**Fig. 1:**
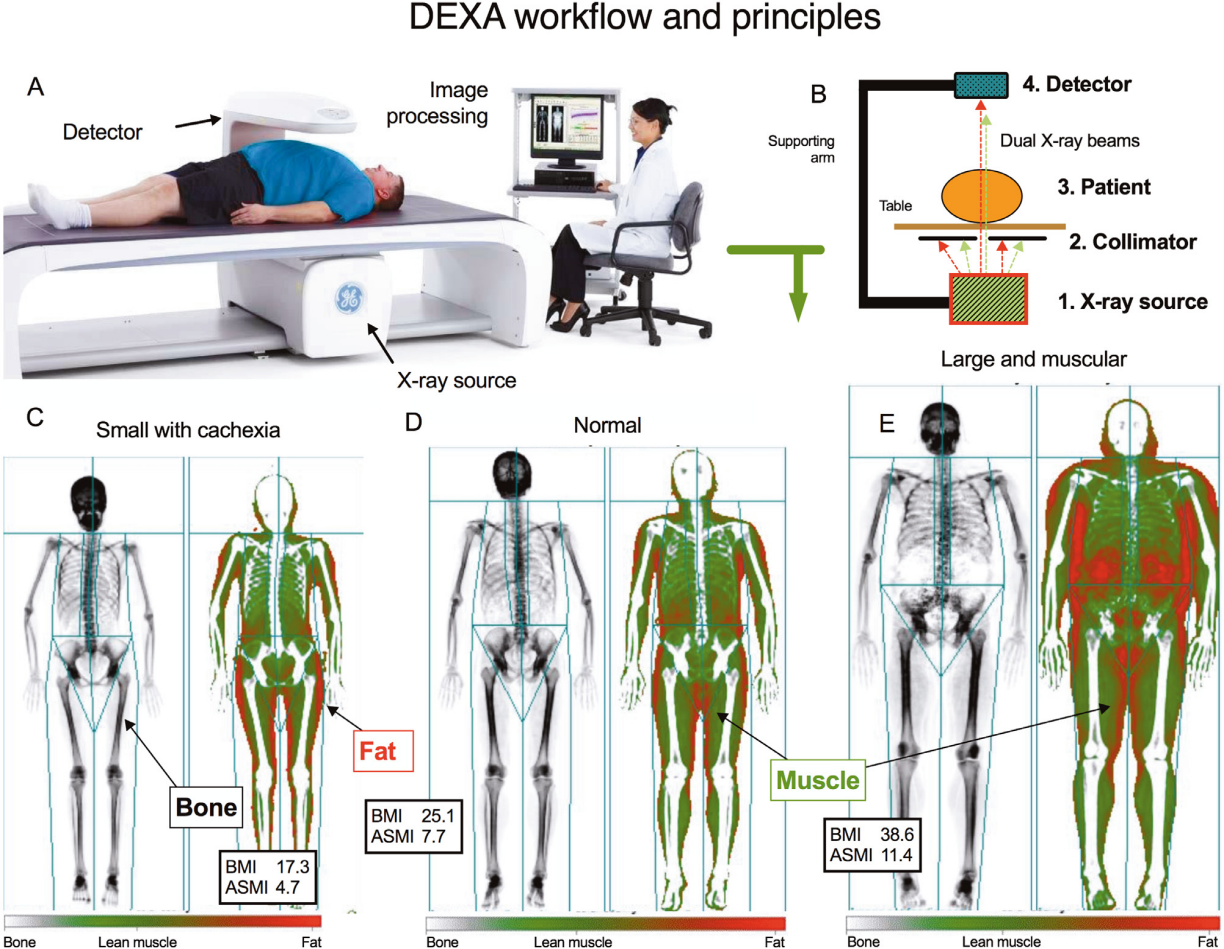
Workflow of DEXA whole body composition analysis. Patients are scanned with a constant potential X-ray generator that produced a beam separated into high and low energy regions (A, image compliments of GE Healthcare). The energy discriminating detector uses the differential attenuation characteristics of these beams to determine the elemental content of tissues using mathematical algorithms (B), separating them into bone (black), fat (red), and lean muscular tissue (green) (C, D, E). Illustrated study patients are small with minimal muscular mass (C, BMI 17·3, ASMI 4·7 kg/m^2^), normal (D, BMI 25·1, ASMI 7·7 kg/m^2^), and large and muscular (E, BMI 11.4, ASMI 38·6 kg/m^2^). DEXA images are scaled to patient height.(For interpretation of the references to color in this figure legend, the reader is referred to the web version of this article.)

The conspicuous discrepancy between eGFR and mGFR is often blamed on unmeasured non-functional factors, especially individual variations in muscle mass or tubular secretion of creatinine. eGFR formulae attempt to infer muscle mass from demographic variables. The muscular source of creatinine is problematical to measure. Reference standards for lean (muscle) mass using CT or MRI are highly correlated with cadaver analysis (*r* = 0·99), but involve either radiation exposure or cost, and tedious regional volumetric analysis [7]. Practical difficulties linking accurate muscle mass measurements with renal functional reference methods have hindered research. The introduction of Dual Energy X-Ray Absorptiometry (DEXA) has overcome that obstacle. Originally developed for bone mineral content (BMC) measurement and osteoporosis diagnosis, DEXA also produces reliable and inexpensive measurements of muscle mass with miniscule radiation exposure (about 0·1μGy). The relative attenuation characteristics of two X-ray energy peaks determine the elemental contents of tissues. Mathematical algorithms then assign pixels based on BMC, fat, or lean muscle which are overlayed onto body regions (Fig. 1). Lean muscle mass by DEXA strongly correlates with CT and MRI reference standards (*R*^2^ 0·86 and 0·96) [8,9], but is much less expensive and time-consuming to perform. DEXA produces total, truncal, appendicular muscle mass measurements, and appendicular skeletal muscle index (ASMI) a relative index of muscularity normalized to height [2] (comparable to BMI scaling for obesity) [10]. ASMI accurately diagnoses sarcopenia and cachexia. In patients with renal failure, lean mass may contribute to bias for MDRD eGFR, however results are conflicted and accurate data sparse [11,12].

We evaluated how muscle mass influences the accuracy of eGFR against isotopic mGFR in kidney transplant recipients using contemporaneous DEXA. eGFR error was significantly affected by muscle mass, formula used (CKD-EPI versus MDRD), trimethoprim blockade of tubular creatinine secretion [13], and the absolute level of mGFR. Muscle mass reduced the performance of CKD-EPI eGFR to predict CKD stage 3 at the extremes of body habitus.

## 2 Methods

### 2.1 Research design and study population

The study design was a single center, cohort study using prospective measurement of muscle mass and renal function. The *a priori* hypothesis tested was creatinine generation from muscle contributes to eGFR error. Consecutive Westmead Hospital renal transplant recipients presenting for their 1-year assessments from January 2018 to March 2020 were screened for contemporaneous mGFR and DEXA studies. Exclusions included patients with amputations, non-concurrent evaluations (>3 months), and extreme body habitus (exceeding the 136 kg DEXA loading capacity). Statistical outliers exceeding the 95%CI were not excluded. The study was conducted in accordance with the Declaration of Helsinki and informed written consent was obtained from patients. Institutional ethics approvals were HREC 2013/12/2·5 (3851) and SSA/14/WMEAD/156. This study was undertaken without external funding. It follows STARD guidelines and includes a checklist (Table S1) [14].

### 2.2 Renal function reference tests

The evaluated index test was 2009 CKD-EPI eGFR [5] adjusted by 0·9408 for isotope dilution mass spectrometry (IDMS) calibration. Serum creatinine was measured by enzymatic colourimetric reaction (ECre_2 assay) using hydrogen peroxide generation [15] (Atellica CH analyser, Siemens Healthcare Diagnostics, Erlangen, Germany). The manufacturer's coefficient of variation (CV) was 1·5% for repeatability and 1·9% for within-lab precision. Our uncertainty values (2xCV%) were 7·8% and 4·2% (76 µmol/L and 508 µmol/L, respectively). All patients were fasted and hydrated prior to testing.

The reference renal function test was isotopic mGFR; determined from the disappearance of radioactivity from two, timed plasma samples after a single injection of technetium-99 m diethylene-triamine-pentaacetic acid (Tc^99m^ DTPA), during stable renal function. This method is highly precise, accurate, reproducible, and independent of volume of distribution of isotope (detailed Text S1) [16].

### 2.3 DEXA and muscular mass measurement

Whole body composition analysis used DEXA (Lunar iDXA, GE Healthcare, Madison, WI) with the manufacturer's software (Encore v16, GE Healthcare, detailed Text S2). The iDXA X-ray generator produces a constant beam, separated by K-edge filtration into high and low energy regions (Fig. 1). Differential attenuation characteristics from the energy discriminating detector reflect elemental tissue content, which is separated into a three-compartment model of bone, fat and lean muscle. Automatic software delineates regions of interest within defined anatomical regions, with occasional manual readjustment. Whole body scans include head, trunk (subdivided into spine, ribs and pelvis), arms and legs. Imaging separation locations for arms and were humeral socket centres and femoral necks, respectively. DEXA lean muscle mass is a composite of non-fat and non-bone tissue. Summated upper and lower limb muscle mass (in kg) was normalized to height [2] to produce ASMI (in kg/m^2^), a proxy for muscularity.

### 2.4 Statistical analysis

eGFR error was the difference between eGFR and mGFR. eGFR error was evaluated by univariable regression, then multivariable analysis after backwards elimination. We used unpaired Student's *t*-test for nominal data and Pearson's correlation for comparisons. Preliminary eGFR performance used Bland and Altman methods for fixed and proportional bias estimates [17]. Subsequent difference plots used mGFR as the independent variable (a better comparison of reference test against suboptimal estimates). Fixed bias (eGFR–mGFR) was mean difference from zero (SD was precision). Proportional bias used the regression coefficient of eGFR error (±SE) against mGFR. Analytical accuracy was reported as percentage eGFR values within 10% (P_10_), and 30% (P_30_) of mGFR. eGFR error scatter plots are presented to illustrate imprecision.

CKD-EPI eGFR (mls/min/1·73 m^2^) is intrinsically normalized to body surface area (BSA), the traditional adjustment of body size for CKD classification. The normalized GFR result is a ratio of function to BSA (which correlated with ASMI, *r* = 0.710, *P*<0.001). We therefore “unindexed” eGFR (to mls/min) to better evaluate relationships with muscle mass, which were highly collinear with BSA and otherwise produce misleading in patients with abnormal body size [18]. Preliminary evaluation found indexing reduced eGFR inaccuracy by 23·5% and 24·0% (ASMI and lean mass versus unindexed values). Sensitivity analyses used (1) residual values from ordinary least-squares linear regression, (2) absolute values to illustrate variation by mGFR, (3) weighted linear regression to adjust for heteroscedasticity, and (4) duplicate analysis using unmodified CKD-EPI eGFR. All multivariable models were verified using collinearity diagnostics. Statistical software for results and figures were GraphPad Prism (version 8.4.3) and Statistical Package for Interactive Data Analysis (SPIDA, Version 6, Macquarie University). Data are expressed as mean±SD, unless stated. Tests were 2-tailed (except for fixed bias versus zero mean), and probabilities <0·05 considered significant.

## 3 Results

### 3.1 Clinical body composition and eGFR results

From 164 patients screened, 37 were excluded (no DEXA for geographical or social reasons, *n* = 17 or non-contemporaneous, *n* = 15, physical constraints, *n* = 5 [large size 2, amputations 2, gamma nail 1]). Included recipients (*n* = 137) were 49·2 ± 14·1 years old, 61·3% male, and 78·8% received a deceased donor transplant (detailed Table S2). Immunosuppression comprised: tacrolimus (92·7%) or cyclosporine (6·6%); mycophenolate (81·8%), azathioprine (8·8%), sirolimus (2·2%), or leflunomide (5·1%); and prednisolone. Recipient's ethnicity was Caucasian (*n* = 94), East Asian (*n* = 23), Indian/South Asian (*n* = 15), Pacific Islander (*n* = 4), and Australian Aboriginal (*n* = 1).

Study population was physically and functionally diverse (Table 1): serum creatinine values ranged from 49 to 352 µmol/L; mGFR, 19 to 135 mls/min; eGFR, 14 to 120 mls/min/1·73 m^2^; weight, 39·9 to 128·0 kg; BMI, 16·9 to 41·9 kg/m^2^ (underweight 5, normal 37, overweight 45, mildly obese 34, severe obesity 16, WHO criteria); and ASMI, 3·82 to 11·41 kg/m^2^ (Australian sarcopenia cutoffs: men<7·0, women<5·5 kg/m^2^) [19]. A patient's eGFR was affected by creatinine generation markers (ASMI, body weight, male recipient) and functional clearance indicators (mGFR, optimal kidney-pancreas donor kidney, and lack of chronic tubular damage) using multivariable regression analysis (*R*^2^ 0·731, Tables 2, S3-S4).

**Table 1:** Key demographic of study population renal functional and body composition parameters by DEXA at 12-months (detailed Table S1). Key: ASMI, appendicular skeletal muscle index; DEXA, dual-energy x-ray absorptiometry; eGFR is estimated GFR.

Renal functional parameters	Mean±SD	Range
Serum creatinine (µmol/L)	119±50	49–352
eGFR (mls/min/1·73m^2^)	65·3 ± 24·0	14–120
Unindexed eGFR (mls/min)	69·3 ± 25·4	17·1–122·8
Indexed mGFR (mls/min/1·73 m^2^)	65·6 ± 22·5	16–130
Isotopic mGFR (mls/min)	70·4 ± 24·8	19–135
**Body composition parameters**		
Total body mass (scales, kg)	77·9 ± 18·7	39·5–130·9
Total lean body mass (kg)	46·9 ± 10·0	26·3–74·3
Appendicular skeletal mass (kg)	20·7 ± 5·5	9·16–37·8
Truncal muscle mass (kg)	26·2 ± 4·9	15·5–41·3
Bone mineral density (kg)	2·45±0·54	1·17–3·85
ASMI (kg/m^2^)	7·40±1·44	3·82–11·41
Fat tissue mass (kg)	31·5 ± 38·2	5·74–59·10
Relative adiposity (% fat)	36·7 ± 9·4	11·3–56·9

**Table 2:** Predictors of transplant eGFR. The unindexed eGFR value was affected by both creatinine generation markers (ASMI, body weight, male recipient) versus renal functional clearance indicators (mGFR, optimal SPK kidney, and lack of chronic tubular damage). Multivariable predictors of eGFR using regression analysis, and included isotopic mGFR and ASMI (R^2^ 0·731, df 126, constant 36·18). Key: ASMI, appendicular skeletal muscle index; DEXA, dual-energy x-ray absorptiometry; SPK, simultaneous pancreas kidney transplant (optimal donated kidneys).

Factor	Coefficient (SE)	P value
ASMI (kg/m^2^)	−8·502 (1·611)	<0·001
Recipient weight (kg)	0·377 (0·115)	0·001
Recipient male	6·215 (2·934)	0·036
SPK (vs kidney)	12·425 (3·128)	<0·001
1-year Banff ct score	−3·301 (1·543)	0·034
mGFR (mls/min)	0·733 (0·060)	<0·001

### 3.2 Performance of eGFR against reference mGFR

**Fig. 2:**
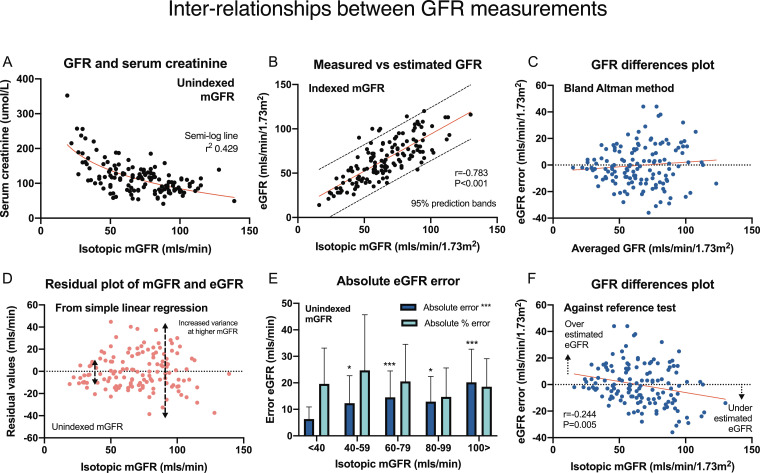
Inter-relationships between GFR estimations and measurements. Panel A illustrates the non-linear relationship between serum creatinine and unindexed measured GFR. Unmodified eGFR correlated with corrected mGFR (B, Pearson correlation). Difference plots of (indexed) eGFR error against averaged corrected eGFR and mGFR (mls/min/1·73 m^2^, Bland-Altman method, Panel C) demonstrate absent fixed and proportional biases. The residual plot against mGFR showed heteroscedasticity and wider variances at higher mGFR levels (Panel D). The absolute value of unindexed eGFR error (eGFR–mGFR, mls/min) increased with higher isotopic mGFR (black columns), however conversion to absolute percentage eGFR error ([*e*GFR–*m*GFR]/mGFRx100%), abrogated the influence of renal function (gray columns, Panel E, * *P*<0·05, *** *P*<0·001 vs <40 mls/min). Corrected GFR error (eGFR-mGFR difference) compared against corrected mGFR (alone) excluded fixed bias, however detected proportional bias with eGFR understimated with greater renal function levels (Panel F).

Serum creatinine and mGFR displayed a characteristic curvilinear relationship (Fig. 2A). Log_e_ creatinine inversely associated with unindexed mGFR (*r*=−0·559, *P*<0·001) and corrected mGFR (*r*=−0·714, *P*<0·001,). CKD EPI eGFR correlated with corrected mGFR (*r* = 0·783, *P*<0·001, Fig. 2B) with good linearity (coefficient±SE 0·833±0·057, *P*<0·001, *R*^2^ 0·612).

The mean indexed and unindexed eGFR errors were −0·26±15·4 mls/min/1·73 m^2^ and −1·164±16·9 mls/min, respectively. Bland-Altman difference plots (versus averaged indexed eGFR and mGFR) excluded fixed or proportional bias (Table 3. Fig. 2C) for CKD EPI, which displayed better diagnostic performance compared with MDRD and Cockcroft-Gault formulae. Unindexed eGFR error was normally distributed (Shapiro-Wilk test 0·989, *P* = 0·923). When indexed GFR error was compared against indexed mGFR, fixed bias was excluded (coefficient±SE −0·263±15·385, *P* = 0·842) however negative proportional bias was detected (−0·167±0·057, *P* = 0·004, Fig. 2F).

**Table 3:** Performance characteristics of unindexed eGFR against reference test mGFR (both in mls/min). Fixed bias is the mean (±SD, the precision) difference between eGFR and mGFR versus zero. Proportional bias is the regression (beta coefficient±SE) slope by mGFR level. Accuracy denotes percentage eGFR results within ±10% and ±30% of mGFR reference (P10% and P30%, respectively). Key: CG, Cockcroft-Gault; CKD EPI, Chronic Kidney Disease EPIdemiology; and MDRD, Modification of Diet in Renal Disease (eGFR formulae).

	FIXED BIAS		PROPORTIONAL BIAS		ACCURACY	
	Mean±SD	P	Coefficient±SE	P	P10 (%)	P30 (%)
**Formula:**						
CKD EPI (mls/min)	−1·164±16·865	0·421	−0·208±0·056	<0·001	28·5%	83·2%
MDRD (mls/min)	−8·561±26·406	<0·001	−0·435±0·084 <0·001	<0·001	18·2%	54·0%
CG (mls/min)	7·900±19·184	<0·001	−0·051±0·067	0·444	33·6%	74·4%

Heteroscadasticity with increasing variance at higher mGFR was apparent in residual and difference plots (Fig. 2C-D, 2F). Absolute unindexed eGFR error similarly increased against mGFR (regression 0·133±0·034, *r* = 0·317, *P*<0·001, Fig. 2E), abrogated by conversion to percentage absolute (|[*e*GFR–*m*GFR]|/mGFRx100%) values (*r* = 0·138, *P* = 0·108) and independent of ASMI (*r*=−0·069, *P* = 0·426, Figure S1).

### 3.3 eGFR error correlated with muscle mass

**Fig. 3:**
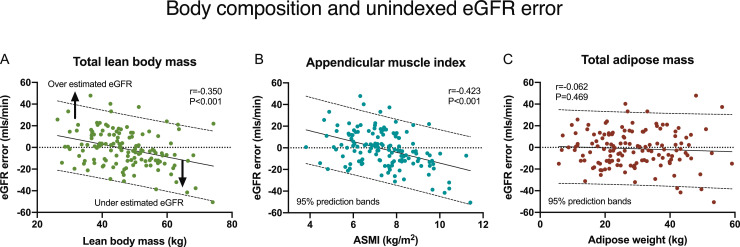
eGFR error was affected by muscular mass. Muscle mass measurements inversely correlated with unindexed eGFR error (eGFR-mGFR) including total lean mass (Panel A) and appendicular skeletal mass index (ASMI, Panel B), resulting in overestimation in cachexic patients and underestimation in muscular recipients. Adipose mass had no effect on eGFR error (Panels C). Key: Pearson correlation coefficients and 95% prediction bands are presented.

All DEXA measurements of muscular mass inversely correlated with eGFR error including: total muscle (*r*=−0·350, *P*<0·001), truncal muscle (*r*=−0·304, *P*<0·001), appendicular skeletal muscle (*r*=−0·370, *P*<0·001) mass, and ASMI (*r*=−0·423, *P*<0·001, Fig. 3, S2). Muscle mass was associated with overestimation of eGFR in cachexia and underestimation in muscular recipients. The eGFR error from muscle was linear and consistent with respective regression coefficients (±SE) per kg of: −0·587±0·135 (total muscle), −1·055±0·285 (truncal), −1·133±0·245 (appendicular mass), and −4·963±0·915 per kg/m^2^ (for ASMI, all *P*<0·001). The false reduction of eGFR was −5·867±1·353 mls/min per 10 kg total lean muscular mass. In contrast, adipose mass and percentage adiposity had no effect (Fig. 3C, S2).

By unweighted multivariable linear regression, ASMI remained a predictor of eGFR error (*P*<0·001), along with trimethoprim use, and recipient age, when controlled for mGFR to adjust for heteroscedasticity; which was confirmed using weighted regression analysis (Tables 4, S5–6).

**Table 4:** Multivariable predictors of unindexed GFR error. Predictors of eGFR error (eGFR-mGFR, mls/min) by linear regression included muscularity and trimethoprim use, along with the level of renal function. Isotopic mGFR (mls/min) was included as a covariate to adjust for heteroscedasticity at higher GFR levels in (unweighted) model 1 (R^2^ 0·279, constant 42·855). Predictors of GFR error using weighted linear regression analysis (model 2, against reciprocal mGFR as the weighting term, R^2^ 0·206, constant 26·309). Key: ASMI, appendicular skeletal muscle index.

Unweighted model 1:		
Factor	Coefficient (SE)	P value
ASMI (kg/m^2^)	−4·364 (0·904)	<0·001
Trimethoprim use	11·046 (4·815)	0·023
Recipient age (years)	−0·219 (0·092)	0·019
mGFR (mls/min)	−0·159 (0·055)	0·004

Weighted model 2:		
Factor	Coefficient (SE)	P value
ASMI (kg/m^2^)	−4·829 (0·864)	<0·001
Trimethoprim use	9·857 (4·892)	0·046

### 3.4 eGFR muscular error varied by formula and trimethoprim use

**Fig. 4:**
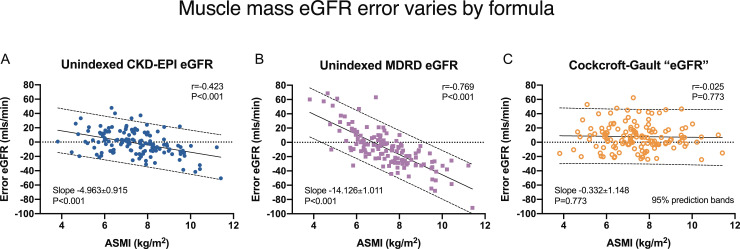
Muscle mass error varies by eGFR formula used. Unindexed eGFR error (eGFR–mGFR) was compared against ASMI, a muscularity marker. The linear regression slope (coefficient±SE) and Pearson's correlation reflect the relative influence of muscle mass on eGFR error. Negative slope coefficients indicate a muscle-dependent underestimation of true mGFR. The error from CKD-EPI eGFR equation was less dependent on muscularity compared with MDRD formula. In contrast, Cockcroft-Gault (which additionally incorporates weight), showed no demonstrable “eGFR” error (derived from creatinine clearance) from muscular mass. Key: ASMI, appendicular skeletal muscle index; CKD-EPI, Chronic Kidney Disease EPIdemiology; MDRD, Modification of Diet in Renal Disease (formulae). Pearson correlation, linear regression coefficient (±SE), and 95% prediction bands are presented.

eGFR error associated with muscularity was highly dependent on the eGFR formula used (Fig. 4). Unindexed eGFR error from CKD EPI was less dependent on ASMI (coefficient −4·963±0·915, *r* = 0·769, *P*<0·001) compared with MDRD (−14·126±1·011, *r* = 0·423, *P*<0·001). Cockcroft-Gault formula displayed no muscle-related error (−0·332±1·148, *r* = 0·025, *P* = 0·773).

Protocol trimethoprim/sulphamethoxazole for Pneumocystis prophylaxis was used in 127 (92·7%) producing a mean eGFR error of −0·25±15·9, increasing to −12·8 ± 24·5mls/min with non-sulpha alternatives (*n* = 10, 7·3%, *P* = 0·023), and independent of confounders (Table S7). Histological tubular atrophy scores, summated Banff ci+ct [20], acute tubular injury, glomerulosclerosis (*n* = 133 biopsies), immunosuppression, corticosteroid exposure, transplant variables, and serum albumin had no effect on error (Table S5).

Random eGFR error was approximated after comprehensive multivariable linear regression analysis. Sequential coefficients of determination (R^2^) were: 0·293 (physical variables including ASMI, *n* = 15); 0·309 (trimethoprim); 0·825 (function including mGFR, *n* = 3); 0·872 (transplant-related, *n* = 16); 0·911 (kidney pathology, *n* = 12). The residual variance was 8·9% (1–0·911).

### 3.5 Serum creatinine independently correlated with muscle mass

**Fig. 5:**
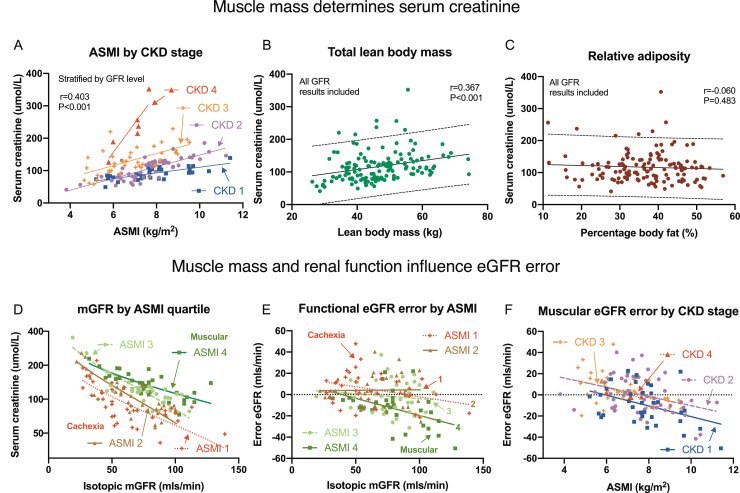
Muscle mass increases serum creatinine concentration. Both the ASMI (in kg/m^2^) and lean muscular mass (in kg) significantly correlated with serum creatinine concentration (Panels A and B), but not with percentage body fat (C). When stratified using unindexed mGFR into CKD stages, serum creatinine still associated with ASMI within each strata of function (A). The plot of serum creatinine against isotopic mGFR when stratified into ASMI quartiles (1st quartile is cachexia, 4th is the most muscular, Panel D) is a family of four curves which are affected by creatinine generation from muscle. eGFR underestimation error was greatest in muscular (ASMI quartile 4) patients with high mGFR levels (E). The negative eGFR error from muscle mass displayed a consistently negatively slope across all CKD stages (panel F). Key: ASMI, appendicular skeletal muscle index; CKD, chronic kidney disease levels were defined as stage 1 (≥90mls/min), 2 (60–89mls/min), 3 (30–59mls/min), and 4 (<30mls/min). Pearson correlation coefficients and 95% prediction bands are presented.

We evaluated the influence of muscularity on serum creatinine, which was log_e_ transformed for statistical normalization. Log_e_ creatinine in unselected patients correlated with total lean (*r* = 0·367, *P*<0·001), truncal (*r* = 0·303, *P*<0·001), appendicular muscle mass (*r* = 0·403, *P*<0·001), and ASMI (*r* = 0·416, *P*<0·001, Table S8); but not percentage fat or total adipose mass (Figs. 5A-D, S2). Multivariable linear regression found serum creatinine was predicted by: muscular inputs including male sex, height [2], and ASMI; and functional markers of mGFR and serum urea (*R*^2^ 0·823, Tables 5, S9).

**Table 5:** Multivariable predictors of serum creatinine**.** Multivariable linear regression analysis of log_e_ serum creatinine concentrations, controlled for renal function using mGFR and serum urea (*n* = 137, R^2^ 0·823, constant 3·602). Key: ASMI, appendicular skeletal muscle index.

Factor	Coefficient (SE)	P value
Recipient sex male	0·074 (0·037)	0·045
Recipient height (m^2^)	0·240 (0·064)	<0·001
ASMI (kg/m^2^)	0·098 (0·012)	<0·001
Serum urea (mmol/L)	0·032 (0·004)	<0·001
mGFR (mls/min)	−0·009 (0·001)	<0·001

### 3.6 ASMI and mGFR exert differential effects on serum creatinine

The inter-relationships between muscular mass, serum creatinine and mGFR, were evaluated by stratification into ASMI quartiles and CKD stages (by mGFR, Tables S10–12). Serum creatinine associated with mGFR within ASMI and mGFR strata, but differed by functional level (Fig. 5A, 5D). Log_e_ creatinine was predicted by ASMI at each CKD level (*P* = 0·015 to <0·001). ASMI and mGFR accounted for substantial variance of creatinine (*R*^2^=0·666), which increased after recipient male, height [2], and serum urea were added (*R*^2^=0·823, Tables 6, S11–14). Within each mGFR level, ASMI associated with serum creatinine (*R*^2^ 0·413–0·805). At high function (GFR≥90 mls/min, *n* = 34), ASMI was the dominant predictor of serum creatinine and could be estimated from creatinine alone, irrespective of mGFR (*R*^2^ 0·471, Table S10c).

**Table 6:** Competing multivariable predictors of serum creatinine by CKD stage. Multivariable predictors of serum creatinine (log_e_ µmol/L) using linear regression to assess relative contributions of renal clearance versus muscular input: using isotopic mGFR (per 10mls/min) and ASMI (kg/m^2^) at different CKD stages. Note that regression coefficient measurement is unit dependent. Key: Coefficient (±SE) is the linear regression slope. Key: ASMI, appendicular skeletal muscle index; CKD, Chronic Kidney Disease.

CKD		N	GFR (10 mls/min)		ASMI (kg/m^2^)	
Stage			Coefficient (SE)	P value	Coefficient (SE)	P value
1.	≥90	34	−0·054 (0·023)	0·027	0·107 (0·018)	<0·001
2.	60–89	55	−0·100 (0·031)	0·003	0·182 (0·018)	<0·001
3	30–59	42	−0·166 (0·038)	<0·001	0·166 (0·025)	<0·001
4	<30	6	−0·195 (0·303)	0·567	0·414 (0·153)	0·074

Within each CKD stage, univariable linear regression of log_e_ creatinine (dependent variable) against ASMI (independent variable) found interactions with muscularity. Unmodifed serum creatinine and mGFR were not correlated in well-functioning kidneys (*R*^2^ CKD1=0·013, CKD2=0·040). This relationship only became significant at mGFR<60mls/min (Table S11). Multivariable linear regression of log_e_ creatinine found differing influences from muscle input (ASMI) relative to renal clearance (mGFR). The regression coefficients increased with dysfunction, but with differing kinetics (Tables 6, S12). Most ASMI muscle-associated eGFR error occurred in muscular recipients of normal kidneys (Fig. 5E).

### 3.7 Diagnostic test performance of eGFR by ASMI quartile

Test performance of unmodified CKD EPI eGFR (mls/min/1·73m^2^) to detect CKD stage 3 (mGFR<60 mls/min/1·73m^2^) was fair: with a sensitivity of 78·0%; specificity 79·5%; and area-under-curve (AUC) of 0·893 (95%CI 0·842–0·944, Figure S5). Extremes of muscularity caused substantial reductions in performance: the sensitivity for CKD3 was 68·4% in the lowest ASMI quartile; and the specificity of 47·4% and positive predictive value (PPV) of 54·5% occurred in 4th quartile (Tables 7, S13).

**Table 7:** Diagnostic performance of eGFR by ASMI quartiles. The test performance of CKD EPI eGFR (unmodified as mls/min/1·73 m^2^) to detect CKD stage 3 (mGFR<60 mls/min/1·73 m^2^) by ASMI muscularity quartile (*n* = 137) Key: PPV and NPV are positive and negative predictive values, respectively. ASMI, appendicular skeletal muscle index where quartile 1 is the least muscular. Results are percentages.

	Sensitivity	Specificity	PPV	NPV
All	78·0	79·5	74·2	82·7
ASMI 1	68·4	93·8	92·9	71·4
ASMI 2	87·5	100	100	90·0
ASMI 3	77·9	80·0	58·3	90·9
ASMI 4	80·0	47·4	54·5	75·0

### 3.8 Surrogate predictors of ASMI and eGFR error

We investigated simple physical markers as potential surrogate predictors for muscularity. ASMI muscularity correlated with body weight (*r* = 0·825, *P*<0·001), male (*r* = 0·480, *P*<0·001), height (*r* = 0·537, *P*<0·001), BMI (*r* = 0·710, *P*<0·001), and BSA (*r* = 0·711, *P*<0·001), but not with recipient age (Fig. S3, Table S14). Multivariable regression found male recipient and weight, independently predicted ASMI (*P*<0·001, *R*^2^ 0·739, Table S15). However, the clinical utility of physical markers to predict error was poor. Unindexed eGFR error correlated with weight (*r* = 0·230, *P* = 0·008), male sex (*P* = 0·028, *t*-test), and BMI (*r* = 0·197, *P* = 0·023, Fig. S4). Multivariable regression found male sex and BMI only weakly predictive of error (*R*^2^ 0·115, Table S16).

## 4 Discussion

KDIGO practice guidelines recommend CKD-EPI eGFR as the preferred initial screening test of kidney function [21]. A recent large systematic review of over 70 eGFR formulae found suboptimal P_30_ accuracy metrics of 60–90% for CKD (10–40% exceeded P_30_); 40–90% for diabetic nephropathy; and 30–90% for kidney transplantation [6]. Poor agreement, lack of concordance, and unpredictable errors were common which remained unchanged by cystatin C use or IDMS calibration. Misclassification of CKD stage was frequent. The authors controversally concluded that eGFR was an unreliable tool to assess renal function in health and disease [6,22,23]. eGFR inaccuracy incorporates systemic bias and imprecision, which are best considered separately. Our study found substantial systemic eGFR error from measured muscle mass that was proportional to lean muscle mass and ASMI quartile, influential across all CKD stages, and dependent on eGFR formula used for calculation. Modest proportional bias underestimated mGFR at higher function (and vice versa), and tubular secretion increased eGFR error by 12·5%. Increasing imprecision which paralleled greater mGFR levels (with dispersion in difference and residual plots) caused by greater variance with higher absolute values, combined with suboptimal predictive capability of serum creatinine in normally functioning kidneys.

Because serum creatinine is closely associated with renal function in the minds of clinicians, the contribution from muscle input is easily overlooked. This is a mistake. Creatinine generation from muscle was an important source of eGFR error which correlated with all measures of muscle mass. eGFR consistently overestimated mGFR in cachexia (reducing sensitivity to 68·4% for CKD3 diagnosis) and underestimated function in the highest ASMI quartile (reducing specificity to 47·4% and PPV to 54·5%). Whole body creatine is equally derived from *de novo* renal synthesis and dietary sources. Following uptake by a high-affinity sarcolemmal transporter into muscle cells, creatine is enzymatically phosphorylated by creatine kinase into phosphocreatine, which comprises 60% of muscular creatine pool. Muscles contain 98% of the body's creatine (type-II “fast-twitch” skeletal tissue more than type-I postural muscles). Creatine is named after κρέας (kréas, Greek for “meat”). Small amounts are found in brain, kidney, and liver. About 1·7% of phosphocreatine dehydrates into creatinine daily, and is released into the water compartment [1]. Lean mass and ASMI proportionally influence serum creatinine concentrations across all CKD stages.

Interestingly, eGFR error associated with ASMI varied according to the formula used for its calculation. The reduced error for CKD-EPI compared with MDRD, could be explained by better muscular estimation from age, sex, and African race variables. CKD-EPI is actually two distinct formulae for male or female subjects, with separate exponents for input demographic predictors. In contrast, Cockcroft-Gault formula (which additionally inputs weight) eliminated muscle mass error (weight strongly correlated with ASMI, *r* = 0·825, *P*<0·001). Discrepancy of eGFR error from muscularity reflects an inadequate compensation of muscular creatinine generation by each formula's variables.

All eGFR error plots displayed substantial scatter or dispersion. Increasing test imprecision paralleled higher renal functional levels, termed heteroscedasticity for difference and residual plots, and was explained by increased variance commensurate with greater absolute mGFR (being eliminated by conversion to absolute percentage values) and suboptimal prediction of mGFR by serum creatinine at extremes of muscularity and function. High level inaccuracy is an old problem for eGFR formulae, and MDRD eGFR was originally reported only to 60 mls/min/1·73 m^2^. To reliably estimate GFR, serum creatinine should be constantly (inversely) related to mGFR across its full range. One unexpected finding was this inter-relationship was inconsistent, and surprisingly weak at higher mGFR (>60 mls/min), where the dominant contributor of creatinine concentration was muscular input (ASMI) rather than functional clearance (mGFR) using multivariable analysis. Large population studies allude to flatter regression slopes of reciprocal creatinine against mGFR in normal kidneys [5,[24], [25], [26]], which possess a greater dynamic range and ability to alter function. The CKD-EPI formula adjusts the serum creatinine exponent above an inflexion point at 62 and 80 µmol/L (female and male, respectively). Poorly-functioning kidneys operate fewer individual nephrons at maximal capacity (hyperfiltration), an display reduced physiological reserve and capability to upscale GFR. Additional imprecision from cachexia and muscularity were amplified at the extremes of renal function (Fig. 5E).

The final potential explanation for test imprecision is random error, which encompasses biological variability and analytic imprecision of index and reference tests [2,6,27]. However, the residual variance after comprehensive multivariable modeling of 47 demographic, muscular, functional, and clinical inputs was only 8·9%. Published within-subject CV values are 4·5% to 8·0% for mGFR (healthy individuals and CKD, respectively); 4·4% (0·7% analytical variation) for serum creatinine; 5·3% for CKD-EPI [28]; and 2·0% for DEXA muscle measurements [29]. Another explanation for eGFR-mGFR differences is methodological mismatch. Both eGFR and mGFR reflect “true GFR”, but calculate results over differing time spans using alternative methodologies. Hour-to-hour GFR acutely varies with hydration, protein loading, and blood pressure, and can precipitously drop to minimal levels with severe hypotension (creatinine and eGFR are initially unchanged but deteriorate after hours or days). Isotopic plasma clearance only calculates GFR during the linear clearance phase following bolus equilibration (i.e. 1 or 2 h) [16]. In contrast, eGFR integrates serum creatinine levels over several days to deduce renal function.

Study strengths include: granular patient data; single center which minimizes test variability; test reference measurements of muscle mass, mGFR, and serum creatinine; and a diverse population with wide physical parameters and functional results. Un-indexed eGFR more accurately evaluates muscle mass interactions with error [18]. Kidney transplant recipients avoid the intrinsic collinearity between muscularity and body size (large, muscular individuals with bigger kidneys produce greater GFR). Routine trimethoprim blockade of tubular secretion without altering mGFR converts serum creatinine into an “ideal” filtration marker [13]. Fixed and proportional biases, precision, and accuracy were analysed separately for clarity. Weakness are relatively small numbers of CKD4 patients (*n* = 6) and trimethoprim use in a transplant cohort, which limits extrapolation of error estimates to the general population, which is likely greater due to variability in inhibited tubular secretion.

We conclude that serum creatinine and eGFR are flawed estimates of true renal function. Systemic error from unmeasured muscle mass, tubular secretion, and proportional bias are added to imprecision at the extremes of function and muscle mass. The suboptimal ability of creatinine to predict clearance in well-functioning kidneys improved with renal dysfunction, where CKD detection is clinically important. Inaccuracies of CKD-EPI eGFR are inextricably linked to the biology of muscular creatinine generation and its relationship to renal clearance. This cannot be easily solved by mathematical re-expression of another similar formula (without weight). Future advances require a fresh approach to eGFR and more research. Cystatin C is an alternative endogenous filtration marker produced by nucleated cells which is independent of muscle mass, diet sex, and age. Combined creatinine–cystatinC eGFR equations perform better than either marker alone [2]. Panels of multiple markers (e.g. low-molecular-weight proteins or metabolites) or novel non-renal serum markers of muscularity may help [2,30]. Until then, clinicians should carefully interpret eGFR results with observed muscularity, reserve accurate mGFR or 24-hour creatinine clearance for selected divergent cases, and use old-fashioned clinical judgment for pateints at the extremes of body habitus.

## Declaration of Interests

All authors declare there are no any conflicts of interest.

## Appendix Supplementary materials

Image, application 1

Image, application 2
