# Supplementary material for: How unmeasured muscle mass affects estimated GFR and diagnostic inaccuracy
Source: eClinicalMedicine. 2020 Dec 1;29-30:100662. doi: 10.1016/j.eclinm.2020.100662 (PMC7788434; doi:10.1016/j.eclinm.2020.100662)
Supplement: Supplementary file 2 [file mmc2.docx]

**SUPPORTING DIGITAL INFORMATION**  Wednesday, 5 August 2020

**Supplementary Figures**

Figure S1. eGFR error against renal function and ASMI.

Figure S2. Unindexed eGFR error and body composition.

Figure S3. Physical determinants of skeletal muscle index.

Figure S4. Physical predictors of unindexed eGFR error.

Figure S5. Prediction of renal impairment by CKD EPI eGFR.

**Supplementary Text**

Text S1. Detailed description of isotopic mGFR technique.

Text S2. Detailed description of DEXA methodology.

**Supplementary Tables**

Table S1. STARD diagnostic accuracy checklist.

Table S2. Comprehensive description of study population and results.

Table S3. Univariable linear regression predictors of unindexed eGFR.

Table S4. Multivariable linear regression predictors of unindexed eGFR.

Table S5. Univariable predictors of unindexed GFR error by linear regression.

Table S6. Multivariable predictors of unindexed GFR error.

Table S7. Effect of trimethoprim use on eGFR error.

Table S8. Univariable correlations of serum creatinine.

Table S9. Multivariable predictors of serum creatinine.

Table S10. Serum creatinine stratified by CKD stage: Descriptive data.

Table S11. Univariable prediction of serum creatinine by ASMI.

Table S12. Multivariable prediction of serum creatinine by ASMI.

Table S13. Diagnostic performance of eGFR by ASMI quartiles.

Table S14. Correlations and univariable predictors of ASMI muscularity.

Table S15. Multivariable predictors of ASMI muscularity.

Table S16. Multivariable predictors of eGFR error for clinical use.

**SUPPLEMENTARY DIGITAL CONTENT FIGURES**

**Figure S1. Unindexed eGFR error and renal function.** The absolute value of unindexed eGFR error (eGFR–mGFR, mls/min) increased with greater isotopic mGFR (Panel A), consistent with observed heteroscedasticity. Conversion to percentage eGFR error ([eGFR–mGFR]/mGFRx100%), resulted in loss of influence of renal function (B), and independence of muscularity using ASMI (C). Key: ASMI, appendicular skeletal muscle index. Dotted lines are 95% prediction bands.

**Figure S2. Unindexed eGFR error and body composition.** eGFR error was affected by muscle but not adipose mass. Muscle mass (in kg) inversely correlated with unindexed eGFR error (eGFR-mGFR): including total lean mass (Panel A), truncal muscle mass (B), and appendicular skeletal mass when measured by DEXA body composition analysis (Panel C), and muscularity using ASMI (D, in kg/m^2^), resulting in overestimation in cachexic patients and underestimation in muscular recipients. There was no effect of adipose mass or relative adiposity for eGFR error (Panels E and F). Key: ASMI, appendicular skeletal muscle index. Pearson correlation coefficients and 95% prediction bands are presented.

**Figure S3. Physical determinants of skeletal muscle index.** The appendicular skeletal muscle index (ASMI, a marker of muscularity) strongly associated with body weight (Panel A), recipient male (B), height (D), BMI (E), and BSA (F), but not with age (Panel C). Key: BMI, body mass index; BSA, body surface area. Dotted lines are 95% prediction bands.

**Figure S4. Physical predictors of unindexed eGFR error.** The unindexed eGFR error (eGFR–mGFR, mls/min) was associated with body weight (r=-0.230, P=0.008, Panel A), male sex (P=0.028 by t test, B), and BMI (r=-0.197, P=0.023, E). However, recipient age (r=-0.093, P=0.281, C), height (r=-0.147, P=0.089, D), and BSA (r=-0.108, P=0.211, F) were not associated with eGFR error. Key: BMI, body mass index; BSA, body surface area. Dotted lines are 95% prediction bands.

**Figure S5.** **Prediction of renal impairment by CKD EPI eGFR.** (A) Prediction of clinically significant renal impaiment (CKD stage 3 defined by mGFR<60mls/min/1.73m^2^) by CKD EPI eGFR (<60mls/min/1.73m^2^) using receiver-operating-characteristic analysis (ROC) for all patients (n=137, Panel A). The AUC was 0.893 (95%CI 0.842-0.944). At 90% specificity to detect CKD 3, eGFR displayed 70.7% specificity. Panel B illustrates the effect of ASMI muscularity quartile (ASMI 1 is least muscular and ASMI 4 is most muscular quartile). Prediction was least at the extremes of muscularity. Panel C a diagnostic matrix comparing diagnostic performance of CKD-EPI eGFR and mGFR (in mls/min/1.73m^2^) to classify CKD stage 3 (<60 mls/min/1.73m^2^, dotted line) with overlaid ASMI quartiles. This illustrates diagnostic misclassification of due to muscle mass (false negative with ASMI/2 and false positive for ASMI3/4). Key: AUC, area under the curve; ASMI, appendicular skeletal muscle index; BMI, body mass index; BSA, body surface area; FN, false negative; FP false positive; ROC, receiver-operating-characteristic (curve); TN, true negative; TP true positive.

**SUPPLEMENTARY DIGITAL CONTENT TEXT**

**Text S1. Isotopic mGFR measurement**

Technetium-99m diethylene-triamine-pentaacetic acid (Tc^99m^ DTPA) was freshly prepared from Tc pertechnetate generator eluate with a lyophilized stannous DTPA kit, according to manufacturers' instructions. The drawn dose of Tc^99m^ DTPA (~60 MBq) was accurately determined using a dose calibrator, itself calibrated daily against a stable reference isotope source. The net activity injected dose was determined by subtracting activity in the plastic syringe before and after intravenous injection, including its extension tubing. The injection site was imaged with a gamma camera to exclude local extravasation.

Heparinised venous blood samples from the contralateral arm were obtained at 2 and 3 hours after injection, with exact times recorded. After centrifugation (10min at 1,000 g), duplicate 1ml plasma aliquots using a calibrated pipette and disposable tips into disposable plastic counting tubes, were assayed for Tc^99m^ activity in an automated well counter. The counter sensitivity was regularly verified by standard dilution against the dose calibrator, and intermittently against a calibrated cobalt-57 source (calibrated at time of the standard dilution above). Tc^99m^ activities were background- and decay-corrected to the time of injection and converted to compatible units.

The plasma clearance of Tc99m was calculated assuming monoexponential kinetics from the formula:

Clearance (ml/min) = Iλ/A_0_,

where I = injected activity, λ = exponential slope of the clearance curve, A_0_ = initial plasma tracer concentration by extrapolation of the clearance curve. Tc^99m^ clearance was reported as ml/min, and additionally corrected for body surface area (BSA) using Dubois’ formula to yield corrected GFR values expressed as mls/min/1.73m^2^

**Text S2. Dual energy X-ray absorptiometry (DEXA)**

Whole body imaging was performed by specialist technicians in the Department of Nuclear Medicine, Westmead hospital using the GE Lunar iDXA, with scans analysed using supplied software (GE Healthcare, Madison, WI).

The iDXA instrument uses a scanning bed located posterior to a supine patient, with a constant potential X-ray generator producing a beam separated by K-edge filtration into high and low energy regions. Anterior to the patient is the energy discriminating detector which uses the differential attenuation of these two energy beams (that are particular to each element and tissue), to calculate whole body and regional composition measurements of bone mineral, fat, and lean soft tissue mass. Assumptions include constant attenuation values for fat and bone mineral, and a minimal effect of hydration on lean tissue. The effective dose equivalent of ionising radiation used is equal or below background levels. Scanning protocols included daily quality control calibration, according to the manufacturer’s instructions.

DEXA scans were analyzed through supplied software (Encore v16, GE Healthcare, Madison, WI) which automatically delineates regions of interest (ROIs) using an algorithm based on internationally agreed areas. Manual readjustments were occasionally necessary using the magnification function of X-rays for bone, and greyscale for designated soft tissue boundaries.

Regional ROIs segmented the whole body scan into standard anatomical regions of head, trunk, two arm and two leg regions, with the trunk subdivided into spine, ribs and pelvis. Imaging separation locations were the centres of the humeral sockets and femoral necks, for arms and legs respectively. Total skeletal muscle mass, and the muscle masses for each limb were then produced. Upper and lower limb skeletal muscle mass results were summated to calculate appendicular skeletal muscle (in kg), which was normalzed to the square of patient height (m^2^) to generate the ASMI, a relative index of appendicular skeletal muscle mass (in kg/m^2^) commonly used as a proxy for muscularity.

Visceral fat mass was calculated by subtraction of abdominal subcutaneous fat from total android fat mass. Subcutaneous fat content was estimated from the lateral abdominal wall thickness with extrapolation to the whole android region, which correlates with CT visceral fat although some underestimation can occur in lean (BMI<25) or thin (values <500gms) patients. The android ROI is automatically placed below the ribs and with its caudal limit to the upper pelvis cut (just above iliac crests) and height calculated as 20% of the distance between the upper pelvis cut and the neck cut (just below the chin). The gynoid upper thigh ROI has a height of twice the android ROI height with its upper boundary distance at 1.5x the android ROI height, below the upper pelvis cut.

The resultant whole body composition analysis included regional and whole body bone mineral density, lean and fat tissue mass and derivative values of bone mineral content, total soft tissue mass, fat free mass, regional/total soft tissue mass ratio, percentage fat, visceral fat mass, total body percentage fat, android (male, “apple”) percentage fat, gynoid (female, “pear”) percentage fat, and the android/gynoid ratio.

**SUPPLEMENTARY DIGITAL CONTENT TABLES**

Table S1. STARD Checklist. Standards for Reporting Diagnostic accuracy studies. Page number is manuscript page, “S” prefix in SDC page number.

|  | **Section & Topic** | **No** | **Item** | **Reported on page #** |
| --- | --- | --- | --- | --- |
|  |  |  |  |  |
|  | **TITLE OR ABSTRACT** |  |  |  |
|  |  | **1** | Identification as a study of diagnostic accuracy using at least one measure of accuracy  (such as sensitivity, specificity, predictive values, or AUC) | 3 |
|  | **ABSTRACT** |  |  |  |
|  |  | **2** | Structured summary of study design, methods, results, and conclusions  (for specific guidance, see STARD for Abstracts) | 3 |
|  | **INTRODUCTION** |  |  |  |
|  |  | **3** | Scientific and clinical background, including the intended use and clinical role of the index test | 7 |
|  |  | **4** | Study objectives and hypotheses | 7 |
|  | **METHODS** |  |  | *7* |
|  | *Study design* | **5** | Whether data collection was planned before the index test and reference standard  were performed (prospective study) or after (retrospective study) | 7 |
|  | *Participants* | **6** | Eligibility criteria | 7 |
|  |  | **7** | On what basis potentially eligible participants were identified  (such as symptoms, results from previous tests, inclusion in registry) | 7 |
|  |  | **8** | Where and when potentially eligible participants were identified (setting, location and dates) | 7 |
|  |  | **9** | Whether participants formed a consecutive, random or convenience series | 7 |
|  | *Test methods* | **10a** | Index test, in sufficient detail to allow replication | 7,9 |
|  |  | **10b** | Reference standard, in sufficient detail to allow replication | 7,8, S7-9 |
|  |  | **11** | Rationale for choosing the reference standard (if alternatives exist) | 5,6 |
|  |  | **12a** | Definition of and rationale for test positivity cut-offs or result categories  of the index test, distinguishing pre-specified from exploratory | 14  KDIGO |
|  |  | **12b** | Definition of and rationale for test positivity cut-offs or result categories  of the reference standard, distinguishing pre-specified from exploratory | 14  KDIGO |
|  |  | **13a** | Whether clinical information and reference standard results were available  to the performers/readers of the index test | 18 |
|  |  | **13b** | Whether clinical information and index test results were available  to the assessors of the reference standard | 18 |
|  | *Analysis* | **14** | Methods for estimating or comparing measures of diagnostic accuracy | 8,9 |
|  |  | **15** | How indeterminate index test or reference standard results were handled | N/A |
|  |  | **16** | How missing data on the index test and reference standard were handled | None missing |
|  |  | **17** | Any analyses of variability in diagnostic accuracy, distinguishing pre-specified from exploratory | 11-13 |
|  |  | **18** | Intended sample size and how it was determined | 7 |
|  | **RESULTS** |  |  |  |
|  | *Participants* | **19** | Flow of participants, using a diagram | 7,9 no diagram |
|  |  | **20** | Baseline demographic and clinical characteristics of participants | 9,10, S12-15 |
|  |  | **21a** | Distribution of severity of disease in those with the target condition | S26-30 |
|  |  | **21b** | Distribution of alternative diagnoses in those without the target condition | n/a |
|  |  | **22** | Time interval and any clinical interventions between index test and reference standard | 7 |
|  | *Test results* | **23** | Cross tabulation of the index test results (or their distribution)  by the results of the reference standard | 25, s31 |
|  |  | **24** | Estimates of diagnostic accuracy and their precision (such as 95% confidence intervals) | 25,26 |
|  |  | **25** | Any adverse events from performing the index test or the reference standard | None; its only a blood test |
|  | **DISCUSSION** |  |  |  |
|  |  | **26** | Study limitations, including sources of potential bias, statistical uncertainty, and generalisability | 10-14,17 |
|  |  | **27** | Implications for practice, including the intended use and clinical role of the index test | 16-17 |
|  | **OTHER INFORMATION** |  |  |  |
|  |  | **28** | Registration number and name of registry | 7 |
|  |  | **29** | Where the full study protocol can be accessed | 18, S7,9 |
|  |  | **30** | Sources of funding and other support; role of funders | 1, 18 |
|  |  |  |  |  |

**Table S2a. Complete summary demographic data.** Mean±SD or number (%).

**Patient data at transplantation:**

Patients (n) 137 patients

Recipient age (years) 49.2±14.1

Recipient male (n, %) 84 (61.3%)

**Causes of end-stage renal failure:**

Glomerulonephritis 60 (43.8%)

Diabetic nephropathy 45 (32.8%)

Interstitial nephritis 3 (0.7%)

Hypertensive sclerosis 5 (3.6%)

Polycystic renal disease 5 (3.6%)

Reflux dysplastic syndrome 12 (8.8%)

Other or unknown 7 (5.1%)

**Transplant related demographics:**

HLA mismatch (of 6) 3.9±1.7

Living donors (n, %) 29 (21.2%)

Kidney / kidney-pancreas 109 / 28

ABO incompatible (n, %) 2 (1.5%)

Any pre-transplant DSA define as any MFI>500

57 (41.6%)

**Induction immunosuppression:**

None 3 (2.2%)

Basilixumab 111 (81.0%)

Anti-lymphocyte globulin 17 (12.9%)

Desensitization / ABOi 2 (1.5%)

**Table S2b. Clinical data at or before biopsy time**

**Early clinical events (<3 months post-transplant):**

Delayed graft function 28 (20.4%)

Early cellular rejection 22 (16.1%)

Early vascular rejection 6 (4.4%)

Early humoral rejection 3 (2.2%)

**Prior events and therapy before 12 month scan**

Pulse IV corticosteroids (%) 50 (36.5%)

Antithymocyte therapy (%) 26 (19.0%)

Prior BK viremia (%) 30 (21.9%)

Prior BKVAN (%) 6 (4.4%)

**Immunosuppression at 12 months**

Tacrolimus / Cyclosporine / nil calcineurin inhibitor

127 / 9 / 1

Mycophenolate / Azathioprine / Lefunomide / mTOR inhibitor /Nil

112 / 12 / 7 / 3 /3

Tacrolimus dose (mg/day) 4.9±3.2 Levels (ng/ml) 14.4±80.6

Cyclosporine dose (mg/day) 227±85 Levels (ng/ml) 172±71

Mycophenolate mofetil (g/day) 1.64±0.49

Prednisolone (mg/day) 9.4±1.6

**Table S2c. Renal functional parameters at 12 months**

**Factor Mean±SD Range**

Serum creatinine (µmol/L) 119±50 49-352

Estimated GFR (mls/min/1.73m^2^) 65.3±24.0 14.0-120.0

Unindexed eGFR (mls/min) 69.3±25.4 17.1-122.8

Corrected mGFR (mls/min/1.73m^2^) 65.6±22.5 16-130

Isotopic mGFR (mls/min) 70.4±24.8 19-135

Corrected GFR error (corrected eGFR- corrected mGFR)

(mls/min/1.73m^2^) -0.26±15.4 -36-44

Unindexed GFR error (estimated-measured GFR)

(mls/min) -1.164±16.9 -50.6-47.8

**Measured physical and calculated body parameters**

Total body mass (scales, kg) 77.9±18.7 39.5-130.9

Recipient height (m) 1.66±0.09 1.45-1.90

Recipient height (m^2^) 2.76±0.30 2.10-3.61

Body mass index (kg/m^2^) 28.22±5.51 16.9-41.9

Body surface area (m^2^) 1.85±0.24 1.31-2.48

**12 month body composition parameters by DEXA**

Total lean body mass (kg) 46.9±10.0 26.3-74.3

Appendicular skeletal mass (kg) 20.7±5.5 9.16-37.8

Truncal muscle mass (kg) 26.2±4.9 15.5-41.3

Bone mineral content (kg) 2.45±0.54 1.17-3.85

ASMI (kg/m^2^) 7.40±1.44 3.82-11.41

Fat tissue mass (kg) 31.5±38.2 5.74-59.10

Visceral adipose mass (kg) 1.55±1.26 0.012-6.33

Relative adiposity (% fat) 36.7±9.4 11.3-56.9

Android/gynoid ratio 1.11±0.28 0.47-1.84

**Table S2d. Detailed matched transplant histopathology**

Biopsies (number) 133

Glomeruli (number) 11.2±6.3

Banff g score 0.04±0.23

Banff i score 0.05±0.33

Banff t score 0.32±0.57

Banff ti score 0.35±0.68

Banff i-IFTA score 1.04±0.94 (n=96)

Banff t-IFTA score 0.58±0.61 (n=97)

Banff v score 0.03±0.22

Banff ptc score 0.01±0.09

Banff ci score 0.95±0.98

Banff ct score 1.09±0.91

Banff cv score 0.77±0.88

Banff cg score 0.04±0.19

Banff ah score 0.43±0.72

C4d scores 0.05±0.32

Tubular necrosis (n, %) 49 (36.8%)

**Table S2e. Principal clinico-pathological diagnosis**

Normal / minimal 48 (36.1%)

Subclinical rejection 3 (2.3%)

Acute rejection 3 (2.3%)

BKVAN 1 (0.8%)

Acute tubular necrosis 2 (1.5%)

IF/TA (nos) 60 (45.1%)

Glomerulonephritis 2 (1.5%)

Chronic antibody rejection 2 (1.5%)

Chronic T cell rejection 9 (6.8%)

Calcineurin nephrotoxicity 3 (2.3%)

**Table S3. Univariable predictors of unindexed eGFR** (in mls/min) using univariable linear regression. R^2^ is the coefficient of determination.

**Factor Coefficient (SE) P value R^2^**

Corrected mGFR

(mls/min/1.73m^2^) 0.844 (0.064) <0.001 0.562

mGFR (mls/min) 0.792 (0.056) <0.001 0.599

Serum creatinine (Ln µmol/L)

-52.264 (3.761) <0.001 0.589

Recipient age (years) -0.605 (0.146) <0.001 0.113

Recipient sex male 5.805 (4.435) 0.193 0.013

Recipient weight (kg) 0.163 (0.116) 0.162 0.014

Recipient height (m^2^) 21.392 (7.054) 0.003 0.003

ASMI (kg/m^2^) -1.164 (1.518) 0.914 0.001

Trimethoprim use 1.502 (8.356) 0.850 0.001

SPK (vs kidney) 20.633 (5.090) <0.001 0.109

Delayed function -15.178 (5.230) 0.004 0.059

Early TCMR -0.669 (5.921) 0.910 0.001

Prior steroids -3.318 (4.506) 0.463 0.004

Prior thymoglobulin -3.925 (5.533) 0.479 0.004

1-year Banff ci score -13.088 (1.977) <0.001 0.251

1-year Banff ct score -13.892 (2.143) <0.001 0.243

Donor age (years) -0.854 (0.099) <0.001 0.357

Donor male -0.621 (4.315) 0.886 0.001

Donor BSA (m^2^) -12.376 (7.511) 0.102 0.002

Donor BMI (kg/m^2^) -1.461 (0.445) <0.001 0.076

Donor LBM (kg) -0.237 (0.193) 0.223 0.011

**Table S4a.** Multivariable predictors of unindexed eGFR using multivariable linear regression analysis including isotopic mGFR (in mls/min) and ASMI. Preferred parsimonious model 1.

**Factor Coefficient (SE) P value**

ASMI (kg/m^2^) -8.502 (1.611) <0.001

Recipient weight (kg) 0.377 (0.115) 0.001

Recipient male 6.215 (2.934) 0.036

mGFR (mls/min) 0.733 (0.060) <0.001

SPK (vs kidney) 12.425 (3.128) <0.001

1-year Banff ct score -3.301 (1.543) 0.034

Constant 36.189 (SE 8.565), P<0.001, R2 0.731, df 126.

**Table S4b.** Multivariable predictors of unindexed eGFR using multivariable linear regression analysis including measured isotopic GFR in mls/min and ASMI). Alternative mixed model 2, with donor age added.

**Factor Coefficient (SE) P value**

ASMI (kg/m^2^) -8.366 (1.651) <0.001

Recipient weight (kg) 0.369 (0.114) 0.002

Recipient male 6.779 (2.911) 0.021

mGFR (mls/min) 0.697 (0.066) <0.001

SPK (vs kidney) 8.049 (3.656) 0.030

1-year Banff ct score -2.625 (1.555) 0.094

Donor age (years) -0.200 (0.097) 0.042

Constant 51.456 (SE 10.940), P<0.001, R2 0.734, df 124.

**Table S4c. Sensitivity analysis. Multivariable predictors of indexed CKD-EPI eGFR.** Multivariable linear regression analysis of eGFR by unmodified CKD-EPI (in mls/min/1.73m^2^) against clinical and morphological parameters. Measured isotopic GFR corrected for BSA was included to control for renal function, meaning resultant predictors are capable of influencing error (n=137). Serum creatinine excluded from analysis (interaction).

**Factor Coefficient (SE) P value**

ASMI (kg/m^2^) -3.391 (0.785) <0.001

Serum urea (mmol/L) -1.435 (0.319) <0.001

Isotopic corrected GFR (mls/min/1.73m^2^)

-0.318 (0.059) <0.001

Constant 57.336 (SE 7.839, P<0.001), R2 0.297, df 133.

**Table S5a. Univariable predictors of unindexed GFR error by linear regression with correlations.** Predictors of unindexed eGFR error “bias” (eGFR-mGFR, mls/min) using univariable linear regression analysis (n=137). Coefficient depend on units of measurement (±SE). R^2^ is the coefficient of determination (the proportion of variance explained by each independent variable, its square root approximates Pearson correlation coefficient).

**Univariable physical and calculated body parameters**

**Factor Pearson P Coefficient (SE) P value R^2^**

Recipient male -0.187 0.030 -6.461 (2.917) 0.028 0.035

Recipient age (years) -0.093 0.281 -0.111 (0.103) 0.280 0.009

Body weight (kg) -0.230 0.008 -0.208 (0.076) 0.007 0.053

Recipient height (m) -0.147 0.089 -27.585 (16.022) 0.087 0.021

Recipient height (m^2^) -0.149 0.084 -8.405 (4.796) 0.082 0.022

Body mass index (kg/m^2^) -0.197 0.023 -0.602 (0.259) 0.021 0.039

Body surface area (m^2^) -0.108 0.211 -7.698 (6.107) 0.210 0.012

**12 month body composition parameters**

Total lean mass (kg) -0.231 0.007 -0.208 (0.075) 0.007 0.053

Appendicular mass (kg) -0.370 <0.001 -0.587 (0.135) <0.001 0.122

Truncal muscle (kg) -0.304 <0.001 -1.055 (0.285) <0.001 0.092

ASMI (kg/m^2^) -0.423 <0.001 -4.963 (0.915) <0.001 0.176

Bone mineral content (kg)

-0.212 0.014 -6.527 (2.593) 0.013 0.045

Fat tissue mass (kg) -0.062 0.469 -0.090 (0.123) 0.468 0.004

Visceral fat mass (kg) -0.169 0.051 -2.262 (1.138) 0.049 0.028

Percentage fat (%) -0.122 0.156 0.220 (0.154) 0.154 0.015

Android/gynoid ratio -0.175 0.043 -10.694 (5.190) 0.041 0.030

**Table S5b. Functional correlates**

**Factor Pearson P Coefficient (SE) P value R^2^**

S. creatinine (µmol/L) -0.275 <0.001 -0.094 (0.28) <0.001 0.076

Creatinine (Ln µmol/L) -0.333 <0.001 -15.092 (3.678) <0.001 0.111

eGFR (mls/min/1.73m^2^) 0.398 <0.001 -0.280 (0.056) <0.001 0.158

eGFR (mls/min) 0.367 <0.001 -0.244 (0.053) <0.001 0.135

Corrected mGFR

(mls/min/1.73m^2^) -0.305 <0.001 -0.172 (0.063) 0.007 0.053

mGFR (mls/min) -0.230 0.008 -0.208 (0.056) <0.001 0.093

Corrected GFR error: eGFR- corrected mGFR

(mls/min/1.73m^2^) 0.956 <0.001 1.048 (0.028) <0.001 0.914

Unindexed GFR Error: estimated-measured

GFR (mls/min) 1 NA 1 NA 1 NA

**Clinical and pathological correlates**

**Factor Pearson P Coefficient (SE) P value R^2^**

Banff ci +ct score 0.014 0.870 0.131 (0.795) 0.870 0.000

Total inflammation -0.068 0.443 -1.641 (2.132) 0.443 0.005

ATN on biopsy 0.029 0.739 1.015 (3.042) 0.739 0.001

Sclerosed glomeruli (%) -0.016 0.857 -0.019 (0.107) 0.857 0.000

Prior IV steroids 0.098 0.254 3.430 (2.990) 0.253 0.010

Antithymocyte therapy 0.220 0.011 9.442 (3.597) 0.010 0.049

Delayed function 0.066 0.443 2.754 (3.579) 0.443 0.004

Prior rejection 0.086 0.320 3.923 (3.925) 0.319 0.007

Trimethoprim use 0.195 0.024 12.565 (5.453) 0.023 0.038

Donor age (years) -0.179 0.038 -0.173 (0.082) 0.037 0.032

Donor male 0.007 0.938 0.228 (2.906) 0.938 0.001

Donor BMI (kg/m^2^) -0.078 0.373 -0.279 (0.312) 0.312 0.006

**Table S6a. Multivariable predictors of unindexed GFR error: Unweighted regression.** Predictors of unindexed eGFR error (eGFR-mGFR, in mls/min) using isotopic mGFR (mls/min), as a covariate to compensate for heteroscedasticity with higher GFR levels was compared to predictors contributing to eGFR error using unweighted multivariable linear regression.

**Factor Coefficient (SE) P value**

Recipient age (years) -0.219 (0.092) 0.019

ASMI (kg/m^2^) -4.364 (0.904) <0.001

mGFR (mls/min) -0.159 (0.055) 0.004

Trimethoprim use 11.046 (4.815) 0.023

Constant 42.855 (9.982) <0.001 R2 0.279, df 132.

**Table S6b. Multivariable predictors of unindexed GFR error: Weighted regression using three models.** Predictors of unindexed GFR error using weighted linear regression analysis (against inverse of unindexed mGFR as the weighting term to compensate for heteroscedasticity with higher GFR results). Recipient age and sex were forced in (model 1), as was trimethoprim (models 2 and 3) which remained. Key: ASMI, appendicular skeletal muscle index.

**Model 1.**

**Factor Coefficient (SE) P value**

ASMI (kg/m^2^) -4.969 (0.995) <0.001

Recipient age (years) -0.137 (0.088) 0.120

Male sex 0.632 (2.817) 0.823

Constant 43.132 (8.418) <0.001 R2 0.197, df 133.

**Model 2.**

**Factor Coefficient (SE) P value**

ASMI (kg/m^2^) -4.911 (0.984) <0.001

Trimethoprim use 0.074 (2.797) 0.979

Recipient age (years) -0.141 (0.087) 0.108

Male sex 0.074 (2.797) 0.979

Constant 33.761 (9.484) <0.001 R2 0.222, df 132.

**Model 3. Final Parsimonious model (with trimethoprim use included)**

**Factor Coefficient (SE) P value**

ASMI (kg/m^2^) -4.829 (0.864) <0.001

Trimethoprim use 9.857 (4.892) 0.046

Constant 26.309 (7.864) <0.001 R2 0.206, df 134.

**Table S7. Effect of trimethoprim use on eGFR error.** Differences in renal functional parameters from protocol use of trimethoprim (with sulphamethoxazole for Pneumocystis prophylaxis, which blocks of tubular secretion of creatinine), using unmodifed CKD-EPI eGFR and unindexed eGFR error terms. (n=137, unpaired t test) Mean±SD.

**Trimethoprim None P**

**Factor**

Number (n, %) 127 (92.7%) 10 (7.3%)

Serum creatinine (µmol/L) 118±50 109±46 0.587

Estimated GFR (mls/min/1.73m^2^) 65.2±23.8 65.9±26.9 0.934

Unindexed eGFR (mls/min) 69.4±25.1 67.9±29.2 0.858

Corrected mGFR (mls/min/1.73m^2^) 64.8±21.7 75.5±30.4 0.139

Isotopic mGFR (mls/min) 69.6±23.8 80.7±23.8 0.175

Corrected GFR error (corrected eGFR-corrected mGFR)

(mls/min/1.73m^2^) 0.45±14.9 -9.80±18.6 0.037

Unindexed GFR error (estimated-measured GFR)

(mls/min) -0.25±15.9 -12.8±24.5 0.023

Absolute unindexed GFR error (estimated-measured GFR)

(% mls/min) 19.0±14.7 27.9±16.9 0.068

**Table S8. Univariable correlations of serum creatinine against clinical and morphological parameters.** Serum creatinine was transformed by its natural logarithm and compared with clinical and morphological parameters using Pearson’s test (r) and univariable linear regression analysis (n=137).

**Factor Pearson P value Coefficient (SE) P value**

**Physical and calculated body parameters**

eGFR (mls/min/1.73m^2^) -0.868 <0.001 -0.013 (0.001) <0.001

eGFR (mls/min) -0.767 <0.001 -0.011 (0.001) <0.001

mGFR (mls/min/1.73m^2^) -0.714 <0.001 -0.012 (0.001) <0.001

mGFR (mls/min) -0.559 <0.001 -0.008 (0.001) <0.001

Serum urea (mmol/L) 0.720 <0.001 0.063 (0.005) <0.001

Recipient sex male 0.302 <0.001 1.411 (0.222) <0.001

Recipient age (years) 0.067 0.435 0.230 (0.062) <0.001

Body weight (kg) 0.289 <0.001 0.006 (0.002) <0.001

Recipient height (cm) 0.274 0.002 0.011 (0.003) 0.001

Recipient height (m^2^) 0.268 0.002 0.333 (0.103) 0.002

Body mass index (kg/m^2^) 0.210 0.020 0.014 (0.006) 0.019

Body surface area (m^2^) 0.256 0.003 0.404 (0.131) 0.002

**Body composition parameters**

ASMI (kg/m^2^) 0.416 <0.001 0.108 (0.020) <0.001

Total lean mass (kg) 0.367 <0.001 0.014 (0.003) <0.001

Appendicular mass (kg) 0.403 <0.001 0.027 (0.005) <0.001

Truncal muscle (kg) 0.303 <0.001 0.023 (0.006) <0.001

BMC (DEXA, kg) 0.316 <0.001 0.215 (0.056) <0.001

Fat tissue mass (kg) 0.104 0.228 0.001 (0.001) 0.227

Visceral fat mass (kg) 0.249 0.004 0.074 (0.025) 0.003

Android/gynoid ratio 0.243 0.005 0.328 (0.113) 0.004

Percentage fat (%) -0.060 0.484 -0.002 (0.003) 0.483

**Table S9a. Multivariable predictors of serum creatinine.** Multivariable linear regression analysis of log_e_ transformed serum creatinine against clinical and morphological parameters, controlled for renal function measured by isotopic mGFR and serum urea to improve sensitivity towards non-functional contributors (n=137). Age and weight fell out, however, height^2^ was chosen and retained in the model as being more statistically stable. Trimethoprim use had no effect. Key: ASMI, appendicular skeletal muscle index.

**Factor Coefficient (SE) P value**

Recipient sex male 0.074 (0.037) 0.045

Recipient height (m^2^) 0.240 (0.064) <0.001

ASMI (kg/m^2^) 0.098 (0.012) <0.001

Isotopic GFR (mls/min) -0.009 (0.001) <0.001

Serum urea (mmol/L) 0.032 (0.004) <0.001

Constant 3.602 (SE 0.152, P<0.001), R2 0.823, df 131.

**Table S9b. Multivariable predictors of serum creatinine: Sensitivity analysis with log_e_ ASMI transformation: Sensitivity analysis.** Multivariable linear regression analysis of log_e_ transformed serum creatinine against clinical and morphological parameters, controlled for renal function using mGFR and serum urea (n=131, R^2^ 0.821, constant 2.927). ASMI was log_e_ transformed to match the data distribution, but results were not greatly altered.

**Factor Coefficient (SE) P value**

Recipient sex male 0.071 (0.037) 0.057

Recipient height (m^2^) 0.238 (0.064) <0.001

Log_e_ ASMI (kg/m^2^) 0.709 (0.087) <0.001

Serum urea (mmol/L) 0.032 (0.004) <0.001

mGFR (mls/min) -0.009 (0.001) <0.001

**Table S10a. Serum creatinine stratified by CKD stage. Detailed description of functional and demographic parameters stratified by CKD stage.** Completed tabulated data of unindexed isotopic mGFR (mls/min) CKD by stages. Mean±SD.

**CKD stage N S. Creatinine mGFR R^2^**

(µmol/L) (±SD, mls/min)

1. ≥90 34 90.9±18.3 101.6±10.9mls/min 0.541

2. 60-89 55 105.8±32.1 75.0±8.4mls/min 0.680

3 30-59 42 136.2±44.7 45.7±9.1mls/min 0.612

4 <30 6 239.3±96.0 25±3.8mls/min 0.907

**CKD N Indexed eGFR Unindexed Indexed error Unindexed**

**stage CKD EPI eGFR eGFR-mGFR Error**

mls/min/1.73 mls/min mls/min/1.73 mls/min

1. ≥90 34 85.1±17.3 92.3±16.4 -6.6±15.4 -9.3±17.9

2. 60-89 55 69.3±19.0 75.1±18.9 -0.2±15.3 0.1±17.5

3 30-59 42 49.4±18.7 49.1±16.4 4.4±14.9 3.3±14.0

4 <30 6 27.0±10.0 26.9±7.6 2.3±6.7 1.9±6.1

**CKD N % Unindexed Error % Absolute unindexed error**

**Stage** (eGFR-mGFR)/mGFR%) |(eGFR-mGFR)/mGFR%)|

1. ≥90 34 -8.56±16.96 15.82±10.26

2. 60-89 55 0.319±23.57 19.17±13.47

3 30-59 42 7.57±29.53 23.64±18.92

4 <30 6 7.17±23.11 16.83±15.99

**Table S10b. Descriptive summary data for demographic and body mass parameters stratified by CKD stage: Detailed data.** Completed tabulated data of unindexed isotopic mGFR (mls/min) CKD by stages. Exploratory analysis. LM is lean muscle mass (by DEXA in kg). Mean±SD.

**CKD N Age Male Relative adiposity**

**Stage**  (years) (%) % fat

1. ≥90 34 42.2±11.7 73.5% 34.3±8.2

2. 60-89 55 49.9±14.1 69.1% 37.3±9.1

3 30-59 42 53.5±14.4 40.5% 38.0±10.3

4 <30 6 51.9±10.3 66.7% 36.0±10.7

**CKD BSA BMI Weight ASMI Total LM**

**Stage** (m2) (kg/m^2^) (kg) (kg/m^2^) (Kg)

1. ≥90 1.90±0.24 28.4±6.0 82.5±19.5 7.78±1.44 51.2±9.7

2. 60-89 1.90±0.23 29.0±5.7 81.7±19.7 7.62±1.43 48.3±9.8

3 30-59 1.78±0.20 27.3±5.0 71.6±15.1 6.87±1.36 42.1±9.1

4 <30 1.78±0.26 26.7±3.9 72.5±16.1 6.80±0.74 43.8±6.9

**Table S10c. Prediction of ASMI by serum creatinine in well functioning kidneys.** Univariable linear regression analysis of ASMI (in kg/m^2^) using log_e_-transformed serum creatinine (µmol/L) in patients CKD stage 1 (mGFR ≥90mls/min, n=34).

**Factor Coefficient (SE) P value**

Creatinine (Ln µmol/L) 4.841 (0.907) <0.001

Constant -13.959 (4.075) 0.002 R^2^ 0.471, df 32.

**Table S11a. Univariable prediction of serum creatinine by ASMI within each CKD stage.** Univariable linear regression analysis of serum creatinine (log_e_ µmol/L, the dependent variable) against appendicular skeletal muscle index (ASMI in kg/m^2^) within each CKD stage (unindexed mGFR, variable, in mls/min). Key: Coefficient (±SE) is regression slope; DF, degree of freedom.

**Dependent variable: Log_e_ serum creatinine (µmol/L).**

**Independent variable: ASMI (kg/m^2^) only.**

**CKD stage Coefficient (SE) P value DF R^2^**

1. ≥90 0.097 (0.019) <0.001 32 0.471

2. 60-89 0.178 (0.020) <0.001 53 0.604

3 30-59 0.160 (0.030) <0.001 40 0.413

4 <30 0.470 (0.116) 0.015 4 0.805

**Table S11b. Univariable predictors of serum creatinine by isotopic mGFR within each CKD stage.** Univariable linear regression analysis of serum creatinine (log_e_ µmol/L) against unindexed isotopic mGFR (independent variable, in mls/min) within each CKD stage, stratified by mGFR (mls/min). Key: Coefficient (±SE) is the linear regression slope; DF, degree of freedom.

**Dependent variable: Log_e_ serum creatinine (µmol/L).**

**Independent variable: mGFR (mls/min) only.**

**CKD stage Coefficient (SE) P value DF R^2^**

1. ≥90 -0.002 (0.013) 0.525 32 0.013

2. 60-89 -0.008 (0.005) 0.142 53 0.040

3 30-59 -0.024 (0.004) <0.001 40 0.437

4 <30 -0.056 (0.024) 0.083 4 0.568

**Table S12a. Multivariable predictors of serum creatinine by CKD stage: Competitive analysis I.** Multivariable predictors of serum creatinine (log_e_ µmol/L) using linear regression to assess the competing contributions (regression coefficients) of unindexed isotopic mGFR (per mls/min and per 10 mls/min) and ASMI (kg/m^2^) at different levels of renal function (unindexed CKD stages). Independent variables were mGFR and ASMI only. Key: Coefficient (±SE) is the linear regression slope.

**Dependent variable: Log_e_ serum creatinine (µmol/L).**

**Independent variables: mGFR and ASMI.**

**CKD N GFR (mls/min) ASMI (kg/m^2^)**

**Stage**  **Coefficient (SE) P value Coefficient (SE) P value**

1. ≥90 34 -0.005 (0.002) 0.027 0.107 (0.018) <0.001

2. 60-89 55 -0.010 (0.003) 0.003 0.182 (0.018) <0.001

3 30-59 42 -0.017 (0.004) <0.001 0.166 (0.025) <0.001

4 <30 6 -0.019 (0.030) 0.567 0.414 (0.153) 0.074

**Dependent variable: Log_e_ serum creatinine (µmol/L).**

**Independent variables: mGFR and ASMI.**

**CKD N GFR (10 mls/min) ASMI (kg/m^2^)**

**Stage**  **Coefficient (SE) P value Coefficient (SE) P value**

1. ≥90 34 -0.054 (0.023) 0.027 0.107 (0.018) <0.001

2. 60-89 55 -0.100 (0.031) 0.003 0.182 (0.018) <0.001

3 30-59 42 -0.166 (0.038) <0.001 0.166 (0.025) <0.001

4 <30 6 -0.195 (0.303) 0.567 0.414 (0.153) 0.074

**Table S12d. Competing multivariable predictors of serum creatinine by CKD stage: Competitive analysis II.** Sensitivity analysis for serum creatinine using total lean muscular mass (in kg) as an alternative muscular marker against mGFR (mls/min), were stratified by CKD stage.

**Dependent variable: Log_e_ serum creatinine (µmol/L).**

**Independent variables: mGFR and total lean mass.**

**CKD N GFR (mls/min) Total lean mass (in kg)**

**Stage**  **Coefficient (SE) P value Coefficient (SE) P value**

1. ≥90 34 -0.005 (0.002) 0.043 0.015 (0.003) <0.001

2. 60-89 55 -0.012 (0.003) <0.001 0.027 (0.003) <0.001

3 30-59 42 -0.018 (0.004) <0.001 0.026 (0.004) <0.001

4 <30 6 -0.019 (0.022) 0.453 0.047 (0.012) 0.031

**Table S12e. Competing multivariable predictors of serum creatinine by CKD stage: Competitive analysis III.** Sensitivity analysis. Multivariable predictors of serum creatinine (log_e_ µmol/L) using linear regression to assess relative contributions of unindexed mGFR (per 10 mls/min) and log_e_ ASMI (log_e_ kg/m^2^) at different levels of renal function (using unindexed CKD stage. Independent variables were only mGFR and ASMI. Key: Coefficient (±SE) is linear regression slope.

**Dependent variable: Log_e_ serum creatinine (µmol/L).**

**Independent variables: mGFR (per 10mls/min ) and log_e_ ASMI.**

**CKD N GFR (10mls/min) Ln ASMI (kg/m^2^)**

**Stage**  **Coefficient (SE) P value Coefficient (SE) P value**

1. ≥90 34 -0.045 (0.024) 0.067 0.799 (0.137) <0.001

2. 60-89 55 -0.096 (0.031) 0.031 1.327 (0.132) 0.003

3 30-59 42 -0.164 (0.038) <0.001 1.139 (0.169) <0.001

4 <30 6 -0.218 (0.305) 0.526 2.676 (1.016) 0.078

**Table S13. Diagnostic performance of eGFR by ASMI quartiles.** The test performance of unmodified CKD EPI and MDRD eGFR values to detect CKD stage 3 (mGFR<60 mls/min or mls/min/1.73m^2^) by ASMI muscularity quartile (ASMI 1 is least muscular quartile, n=137). Key: PPV and NPV are positive and negative predictive values, respectively.

**1. CKD EPI eGFR (unmodified as mls/min/1.73m^2^)**

Corrected eGFR for corrected CKD stage 3 (mGFR<60 mls/min/1.73m^2^)

**Sensitivity Specificity PPV NPV**

All 78.0 79.5 74.2 82.7

ASMI 1 68.4 93.8 92.9 71.4

ASMI 2 87.5 100 100 90.0

ASMI 3 77.9 80.0 58.3 90.9

ASMI 4 80.0 47.4 54.5 75.0

**2. MDRD eGFR (as mls/min/1.73m^2^)**

MDRD eGFR for CKD stage 3 (mGFR<60 mls/min/1.73m^2^)

**Sensitivity Specificity PPV NPV**

All 78.0 74.4 69.7 81.7

ASMI 1 68.4 93.8 92.9 71.4

ASMI 2 87.5 94.4 93.3 89.5

ASMI 3 77.8 72.0 50.0 90.0

ASMI 4 80.0 42.1 52.2 72.7

**Table S14. Correlations and univariable predictors of muscularity.** The appendicular skeletal muscle index (ASMI) was compared with multiple physical, calculated, and DEXA-measured body composition parameters using Peason’s correlation (r) test and univariable linear regression analysis (n=137).

**Factor Pearson P value Coefficient (SE) P value**

**Physical and calculated body parameters**

Recipient sex male 0.480 <0.001 1.411 (0.222) <0.001

Recipient age (years) -0.067 0.439 -0.007 (0.222) 0.438

Creatinine (log_e_ µmol/L) 0.416 <0.001 1.607 (0.302) <0.001

eGFR (mls/min/1.73m^2^) -0.248 0.004 -0.015 (0.005) 0.003

mGFR (mls/min) 0.278 <0.001 0.016 (0.005) <0.001

GFR error (mls/min) -0.423 <0.001 -0.036 (0.007) <0.001

eGFR error (mls/min) -0.355 <0.001 -0.033 (0.008) <0.001

Body weight (kg) 0.825 <0.001 0.063 (0.004) <0.001

Recipient height (cm) 0.537 <0.001 0.086 (0.012) <0.001

Recipient height (m^2^) 0.545 <0.001 2.620 (0.347) <0.001

Body mass index (kg/m^2^) 0.710 <0.001 0.185 (0.016) <0.001

Body surface area (m^2^) 0.711 <0.001 4.323 (0.368) <0.001

**12 month body composition parameters**

Total lean mass (kg) 0.886 <0.001 0.127 (0.006) <0.001

Appendicular mass (kg) 0.940 <0.001 0.245 (0.008) <0.001

Truncal muscle (kg) 0.480 <0.001 0.227 (0.016) <0.001

BMC (DEXA, kg) 0.702 <0.001 1.846 (0.161) <0.001

Fat tissue mass (kg) 0.373 <0.001 0.014 (0.003 <0.001

Visceral fat mass (kg) 0.517 <0.001 0.591 (0.084) <0.001

Android/gynoid ratio 0.435 <0.001 2.271 (0.405) <0.001

Percentage fat (%) 0.076 0.380 0.012 (0.013) 0.379

**Table S15. Multivariable predictors of muscularity.** Multivariable linear regression analysis of appendicular skeletal muscle index (ASMI in kg/m^2^ as the dependent variable) was compared against optimal body composition variables (n=137). Recipient weight was good or better than BMI or BSA.

**Factor Coefficient (SE) P value**

Recipient sex male 0.748 (0.273) <0.001

Recipient weight (kg) 0.057 (0.004) <0.001

Constant 2.429 (SE 0.273, P<0.001), R2 0.739, df 134.

**Table S16.** **Multivariable predictors of unindexed GFR error restricted to clinically-available markers: Sensitivity analysis.** Multivariable linear regression analysis of unindexed eGFR used predictors available in the nephrology clinic (excluding mGFR and ASMI) for practical modelling. Weight could substitute for BMI in the model, but height and height^2^ were of no additional value. Key: BMI, body mass index.

**Factor Coefficient (SE) P value**

Male sex -6.757 (2.821) 0.018

BMI (kg/m^2^) -0.593 (0.250) 0.019

Trimethoprim 13.524 (5.279) 0.012

Constant 7.188 (8.730) <0.001 R^2^ 0.115, df 133.
